# Reliability of the Grading System for Voiding Cystourethrograms in the Management of Vesicoureteral Reflux: An Interrater Comparison

**DOI:** 10.1155/2016/1684190

**Published:** 2016-03-16

**Authors:** Süleyman Çelebi, Seyithan Özaydın, Cemile Beşik Baştaş, Özgür Kuzdan, Cankat Erdoğan, Mehmet Yazıcı, İsmail Caymaz, Serdar Sander

**Affiliations:** ^1^Department of Pediatric Urology, Kanuni Sultan Suleyman Education and Research Hospital, 34300 Istanbul, Turkey; ^2^Department of Pediatric Surgery, Kanuni Sultan Suleyman Education and Research Hospital, 34300 Istanbul, Turkey; ^3^Department of Pediatric Radiology, Baskent University Faculty of Medicine, Istanbul, Turkey

## Abstract

*Aim*. Vesicoureteral reflux (VUR) is one of the most common conditions seen in pediatric urology. Fortunately, there are many treatment options for this disorder. The grading system for VUR varies among doctors, and the literature on its reliability is sparse. Here, we assessed the effectiveness of the current VUR grading system.* Methods*. A series of 40 voiding cystourethrogram (VCUG) studies were selected. Four pediatric urologists (PU) and four pediatric radiologists (PR) independently graded each VCUG and then agreed on a uniform interpretation. For statistical analysis the intraclass correlation coefficient (ICC) was applied to assess interrater agreement.* Results*. ICC values ranging from 0.82 to 0.88 reflected the strong reliability of VCUG for grading cases of VUR among pediatric urologists and radiologists as separate groups, and the reliability between the two groups was also good, as indicated by an ICC of 0.89. Despite the high ICC, disagreement existed between raters; the lowest agreement was associated with middle grades (III and IV).* Conclusions*. The interrater reliability of the international grading system for VUR was high but imperfect. Thus, grading differences at middle grades can profoundly influence the type of treatment pursued.

## 1. Introduction

Vesicoureteral reflux (VUR), defined as retrograde flow of urine from the bladder back up the ureter into the kidney, is diagnosed in 30% to 40% of children who present with urinary tract infections (UTIs). It is a congenital condition that may resolve or improve over time [1]. Because of the relatively high prevalence of renal scarring, it is prudent to understand how to identify VUR, potential problems associated with chronic VUR, and the most effective therapeutic strategies.

Currently, the standard test for diagnosing VUR is the voiding cystourethrogram (VCUG), which is also used to classify the severity of reflux. It is very important that physicians reach a consensus regarding the stages of VUR, because staging determines whether each child should simply be closely observed, receive prophylactic antibiotics, or undergo endoscopic treatment or surgery. However, the reliability of interrater reflux grading has largely been ignored in the literature [2]. Thus, the goal of this study was to investigate the accuracy of the current staging system by assessing interrater reliability among radiologists and pediatric urologists.

## 2. Methods

After approval by the local institutional review board, we recruited children with primary VUR after their occurrence of UTI between 2012 and 2013. Patients who underwent VCUG and had bladder exstrophy or other abnormalities including ectopic ureterocele and ureteral duplication were excluded from the study. The VCUGs of these patients were shown to four pediatric urologists and four radiologists who are experienced in uroradiology; these physicians were asked to grade VUR (from I to V) according to the international system of radiographic grading of vesicoureteric reflux [3]. The interrater reliability between the pediatric urologists and radiologists was given a grade of 0–5. Reviewers were blinded to the report and other readings. The responses from the pediatric urologists and radiologists were compared, and discrepancies were adjudicated. For statistical analysis, the intraclass correlation coefficient (ICC) was used to calculate interrater reliability. ICC > 0.72 indicated adequate reliability, whereas an ICC > 0.8 indicated almost perfect reliability.

## 3. Results

Unilateral VUR was diagnosed in 40 children (28 girls, 12 boys). The median age was 12 months, and most children (91%) were enrolled after their recurrent UTI. The UTI prior to enrollment was both febrile and symptomatic in 24 children, only febrile in 7, and only symptomatic in 9 children. All cases of VUR were diagnosed using VCUG. In total, 40 VCUGs were reviewed by 4 pediatric urologists and 4 radiologists, yielding a total of 320 observations. Among the pediatric urologists, ICC scores were consistently > 0.8, between 0.83 and 0.87, indicating good reliability (Table 1). Among the radiologists' evaluations, the ICC scores were consistent, ranging from 0.82 to 0.85 (Table 2), indicating the reliability of VCUG grading in this group of physicians. The ICC values demonstrated the strong reliability of VCUG for grading VUR cases among pediatric urologists and radiologists, and the interrater agreement was 0.88 (Table 3).

A closer inspection of scoring among pediatric urologists and radiologists revealed significant discrepancies between grades III and IV. For pediatric urologists, one rater graded 7 cases (17.5%) as III, while the other raters graded 16 cases (40%) as III. Similar ratings were given by the radiologists; 1 radiologist graded 6 cases (15%) as III, while the others graded 15 cases (37.5%) as III (Table 3). The same type of discrepancy also occurred for cases given a grade of IV. For pediatric urologists, 10 cases (25%) were graded as IV by one rater, while another rater graded 20 cases (50%) as IV. The radiologists used similar grades; 1 radiologist graded 9 cases (22.5%) as IV, while the other radiologist graded 17 cases (42.5%) as IV (Table 4).

Although ICC scores were consistently > 0.7 (0.73), there was also a discrepancy between cases which were graded III among the pediatric urologists and radiologists. The pediatric urologists rated 47 renal cases as grade III, while the radiologists scored 39 renal cases as grade III (Table 4).

## 4. Discussion

VUR is present in 30% to 40% of children with a UTI and is associated with a higher risk of renal scarring. Reports from the 1960s and 1970s, when VUR was less frequently recognized, revealed that renal scarring due to VUR was the etiology of 50% of hypertension cases and 30% of end-stage renal disease (ESRD) cases in children [4]; therefore, the current standard of care includes imaging to assess the presence and extent of VUR [5]. Due to the risk of renal scarring, common clinical practice guidelines now state that VCUG is recommended after the first episode of febrile UTI for all children, depending on sex, age, and clinical presentation [6, 7]. The advantage of this method is the ability to grade reflux severity using the widely accepted 5-level International Scale [5]. The majority of children affected by this condition have low-grade VUR (grades I-II); the strategy depends on the hypothesis that reflux, especially VUR of grade III or greater, increases the risk of recurrent UTIs and renal scarring [8, 9]. Thus, grading must be accurate so that reflux of grade III or higher can reliably be distinguished from lower grade reflux to guide the decision [10, 11].

Craig et al. [12] reported near perfect agreement (kappa 90% to 91%) when three radiologists separately graded contrast VCUGs. However, Kronemer et al. [13] reported that there was divergent grade interpretation in 20 of 39 patients with VUR when 2 radiologists separately read the studies. Metcalfe et al. [2] also analyzed reflux grades and concluded that although the overall VUR grading of VCUGs was shown to be reliable, agreement was highest at the extremes of the scale (grades I and V); scoring discrepancies were more common at the middle grades (II–IV). One of the last issues from Greenfield et al. reported that there was divergent grade interpretation in 9 of 61 ureters initially assessed as middle grade (15%). Of these 9 discrepancies 7 (78%) were adjudicated to the higher grade. Greenfield et al. concluded that discrepancies in the assessment of intermediate grade vesicoureteral reflux were noteworthy and added in particular that there was considerable disagreement in the evaluation of intermediate grades of reflux [14].

Our study found that, among groups of pediatric urologists and radiologists, and also in comparisons between the two groups, the ICC value was close to 0.9, indicating reliable grading. There was disagreement regarding grade in up to 20% of individual ureteral readings. We found that most discrepancies concerned VUR of grades III and IV, because both grades subjectively depend on the appearance of the renal calyx without numerical values being taken into account. This finding suggests that in clinical practice there may be reasonable doubt when categorizing children with reflux to grade III. This issue becomes especially important if one is not going to treat a child with grade III reflux with prophylaxis or perform follow-up imaging.

The grading of reflux using the International Scale remains the major means of categorizing patients and determining treatment [15]. Although currently there are different objective parameters such as bladder volume [16, 17] and ureteral diameter [18] to determine the resolution rates, still nearly common principle is using VUR grade for deciding the treatment. In some cases, a discrepancy exists between the degree of dilatation of the pyelocalyceal system and the distal ureter which in turn makes grading difficult. Resolution rate of grade 4 is less than grade 3 and so will require more surgery [19, 20]. Therefore, grade 3 and 4 distinction is important but as our results demonstrated, VUR grade often varies on the middle grades (3 and 4) depending on the observer.

Confusion in this staging system arises from the fact that the current 5-level grading system cannot easily be applied to VCUGs, which only include characteristics of four stages: stage 1, affecting the ureter but reflux does not reach the renal pelvis; stage 2, affecting the ureter with reflux reaching the renal pelvis; stage 3, affecting the renal calyceal system; and stage 4, gross dilatation and kinking of the ureter with papillary impressions no longer visible. More objective and quantitative data are required to divide VCUG findings into five stages.

While the current VUR grading system focuses primarily on the radiographic appearance of the upper tract, VUR grading may be difficult when discrepancies exist between the degree of dilation of the pyelocalyceal system and the ureter. McMillan et al. [16] and Knudson et al. [17] demonstrated bladder volume at the onset of reflux as another independent factor affecting resolution rates, concluding that bladder volume is an objective value that could be utilized to provide prognostic information regarding the spontaneous resolution of vesicoureteral reflux (VUR) in patients. This knowledge could benefit unresolved cases where there were noteworthy discrepancies in the assessment of the grade of VUR. The present study by Cooper et al. [18] revealed the ureteral diameter and concluded that resolution may be more accurately predicted by the appearance of the distal ureter and concluded that ureteral diameter ratio (UDR) correlates with grade of reflux and proves more predictive of the ultimate clinical outcome in children with primary reflux than grade alone [18].

A lack of consensus on VUR grade staging may lead to a discrepancy in clinical decisions. The poor agreement on moderate grades may stem from differences in judging dilation of the calyceal system. The combined review of images by multiple reviewers, or improving the current grading system with including numerical value of calyceal dilatation, may help reduce grading discrepancies.

A discrepancy exists between the degree of dilatation of the pyelocalyceal system and renal parenchymal damage which in turn makes grading difficult [21, 22]. This may reflect situations where the degree of upper tract dilation and parenchymal damage upon which VUR grade is assigned is not well matched [23]. At VUR diagnosis using a combination of grade, age, volume at reflux onset, and presentation with a history of prenatal hydronephrosis and renal parenchymal damage improves predictive ability regarding early reflux resolution. Combining these individual factors may improve the decision-making process regarding reflux management.

In conclusion, VCUG has long been a mainstay of the diagnosis and grading of VUR, and our study confirmed the reliability of this method. However, discrepancies arise in grading abnormalities of the calyceal system seen on VCUGs, which could greatly impact the treatment method used. The combined review of images by multiple reviewers, or improving the current grading system, may help reduce grading discrepancies.

## Figures and Tables

**Table 1:** Assessment of VUR grading among pediatric urologists.

Pediatric urologists (PU)	Intraclass correlation coefficient	95% confidence interval	
		Lower bound	Upper bound
Pediatric urologist 1	0.86	0.79	0.89
Pediatric urologist 2	0.85	0.80	0.88
Pediatric urologist 3	0.83	0.81	0.88
Pediatric urologist 4	0.87	0.83	0.88

**Table 2:** Assessment of VUR grading among pediatric radiologists.

Radiologists	Correlation coefficient	95% confidence interval	
		Lower bound	Upper bound
Radiologist 1	0.84	0.82	0.88
Radiologist 2	0.83	0.81	0.89
Radiologist 3	0.85	0.83	0.89
Radiologist 4	0.82	0.79	0.88

**Table 3:** Agreement between PR and PU on VCUG assessments.

	Intraclass correlation coefficient	95% confidence interval	
		Lower bound	Upper bound
Pediatric urologists	0.887	0.879	0.937
Radiologists	0.854	0.839	0.864

**Table 4:** Adjudication of discrepancies in VUR grade among pediatric urologists (PU) and pediatric radiologists (PR).

Grade	PU1	PU2	PU3	PU4	PR1	PR2	PR3	PR4
1	4 (10%)	3 (7.5%)	3 (7.5%)	4 (10%)	5 (12%)	3 (7.5%)	4 (10%)	3 (7.5%)
2	4 (10%)	5 (12.5%)	4 (10%)	3 (7.5%)	6 (15%)	5 (12.5%)	4 (10%)	3 (7.5%)
3	9 (22%)	15 (37.5%)	7 (17.5%)	16 (40%)	6 (15%)	15 (37.5%)	8 (20%)	10 (25%)
4	14 (35%)	10 (25%)	20 (50%)	10 (25%)	16 (40%)	9 (22%)	15 (37%)	17 (42%)
5	9 (22%)	7 (17%)	6 (15%)	7 (17%)	7 (17%)	8 (20%)	9 (22.5%)	7 (17.5%)
